# Integrity of the Prefronto-striato-thalamo-prefrontal Loop Predicts Tai Chi Chuan Training Effects on Cognitive Task-switching in Middle-aged and Older Adults

**DOI:** 10.3389/fnagi.2020.602191

**Published:** 2021-02-15

**Authors:** Meng-Tien Wu, Pei-Fang Tang, Wen-Yih Isaac Tseng, Yung-Chin Hsu, Yu-Jen Chen, Joshua O. S. Goh, Tai-Li Chou, Yu-Kai Chang, Susan Shur-Fen Gau, Ching Lan

**Affiliations:** ^1^School and Graduate Institute of Physical Therapy, College of Medicine, National Taiwan University, Taipei, Taiwan; ^2^Yonghe Cardinal Tien Hospital, Taipei, Taiwan; ^3^Graduate Institute of Brain and Mind Sciences, College of Medicine, National Taiwan University, Taipei, Taiwan; ^4^Department of Physical Medicine and Rehabilitation, National Taiwan University Hospital, Taipei, Taiwan; ^5^Neurobiology and Cognitive Science Center, National Taiwan University, Taipei, Taiwan; ^6^Center for Artificial Intelligence and Robotics, National Taiwan University, Taipei, Taiwan; ^7^College of Medicine, Institute of Medical Device and Imaging, National Taiwan University, Taipei, Taiwan; ^8^Department of Psychology, College of Science, National Taiwan University, Taipei, Taiwan; ^9^Department of Physical Education, National Taiwan Normal University, Taipei, Taiwan; ^10^Institute for Research Excellence in Learning Sciences, National Taiwan Normal University, Taipei, Taiwan; ^11^Department of Psychiatry, National Taiwan University Hospital, Taipei, Taiwan

**Keywords:** diffusion spectrum imaging, executive function, mind-body exercise, randomized controlled trial, white matter

## Abstract

Tai Chi Chuan (TCC) exercise has been shown to improve cognitive task-switching performance in older adults, but the extent of this positive effect varies among individuals. Past research also shows that brain white matter integrity could predict behavioral gains of cognitive and motor learning. Therefore, in this randomized controlled trial (NCT02270320), we examined whether baseline integrity of three target white matter tract groups was predictive of task-switching improvement after 12-week TCC training in middle-aged and older adults. Thirty-eight eligible participants were randomly assigned to a TCC group (*n* = 19) and a control group (*n* = 19). Cognitive task-switching and physical performances were collected before and after training. Brain diffusion spectrum MR images were acquired before training and the general fractional anisotropy (GFA) of each target white matter tract group was calculated to indicate baseline white matter integrity of that group. Correlation and regression analyses between these GFAs and post-training task-switching improvement were analyzed using adjusted *p*-values. After 12 weeks, significant task-switching and physical performance improvements were found only in the TCC group. Moreover, higher baseline GFA of the prefronto-striato-thalamo-prefrontal loop fibers (*r* = −0.63, *p* = 0.009), but not of the prefronto-parietal/occipital (*r* = −0.55, *p* = 0.026) and callosal (*r* = −0.35, *p* = 0.189) fiber groups, was associated with greater reductions of task-switching errors after the TCC training. Multiple regression analysis revealed that baseline GFA of the prefronto-striato-thalamo-prefrontal loop fibers was the only independent white matter integrity predictor of task-switching error reductions after TCC training (β = −0.620, adjusted R^2^ change = 0.265, *p* = 0.009). These findings not only highlight the important role of baseline integrity of the prefronto-striatal circuits in influencing the extent of positive cognitive task-switching effects from short-term TCC training, but also implicate that preserving good white matter integrity in the aging process may be crucial in order to gain the best cognitive effects of exercise interventions.

## Introduction

Cognitive task-switching, the shifting between cognitive tasks or mental sets (Monsell, 2003), has been shown to be susceptible to age-related declines (Kray and Lindenberger, 2000; Reimers and Maylor, 2005). These declines, however, are modifiable through exercises. Evidence shows that Tai Chi Chuan (TCC) exercise training may reduce such declines in many, although not all, older adults (Tao et al., 2016; Wu et al., 2018). TCC exercise is a multi-component exercise known to improve physical and also cognitive functions, among which cognitive task-switching improvement is frequently reported (Chang et al., 2014). When performing a series of TCC movements, one has to smoothly shift from one postural form to another as if doing sequential motor task-switching drills. Practicing motor-switching drills during TCC training may enhance brain processes generally involved in task-switching, thus allowing for transferring improvement in motor task-switching to cognitive task-switching (Wu et al., 2018).

It remains unclear, however, why some older adults benefit more from TCC training than others. Several functional MRI studies have reported individual differences in TCC training-induced brain plasticity that is associated with differential corresponding extents of cognitive improvement. In particular, individuals with greater TCC training-related gains in task-switching showed greater enhancement of frontal neural engagement during task-switching (Wu et al., 2018), greater increase in the amplitude of low frequency signal fluctuations in the middle frontal gyrus at rest (Yin et al., 2014), or stronger resting-state functional connectivity between the medial prefrontal cortex and the medial temporal lobe (Li et al., 2014). These previous findings shed light on brain functional changes after TCC training, reflecting possible mechanisms of task-switching improvement after TCC training. However, one of the key issues that remain is whether we can predict who might benefit from TCC training the most before the training starts.

Previous literature shows that structural integrity of brain white matter prior to training is a key determinant of behavioral gain after cognitive or motor training. de Lange et al. (2016) found that the white matter integrity of the anterior corpus callosum, thalamic radiation, and inferior fronto-occipital fasciculus tracts predicted memory improvement after memory training in older adults. Bennett et al. (2011) and Song et al. (2012) reported that higher baseline white matter integrity of specific prefronto-striatal tracts was associated with better motor sequence learning in both young and older adults (*r* = 0.55–0.82). In addition, older adults who have better integrity of the left superior longitudinal fasciculus, inferior longitudinal fasciculus, inferior fronto-occipital fasciculus, splenium-parietal callosal fibers (CFs), the genu region of the corpus callosum, or the prefronto-striato-thalamo-prefrontal (PSTP) loop, present better task-switching performance (Madden et al., 2009; Gold et al., 2010; Ystad et al., 2011; Serbruyns et al., 2016; Jolly et al., 2017). We note that all these fiber tracts have connections with either the prefrontal cortex, the parietal cortex, or the basal ganglia, i.e., the brain regions implicated in cognitive switching (Leh et al., 2007, 2010). Intriguingly, degradation of these white matters has also been observed in patients with Parkinson's disease (Taylor et al., 2018; Lenka et al., 2020), a neurological disorder disrupting functional connectivity and metabolism of the dopaminergic fronto-striatal pathways (Wichmann and DeLong, 2013; Monnot et al., 2017; Chu et al., 2019). Patients with Parkinson's disease often present poor task-switching performance (Cools et al., 2001; Cameron et al., 2010). In patients with Parkinson's disease and psychosis, poorer white matter integrity is associated with poorer task-switching performance (Lenka et al., 2020). The converging evidence from research on motor sequence learning and on basal ganglia disorders led us to speculate that baseline integrity of task-switching related white matter tracts may influence the amount of task-switching improvement after TCC training in the older population.

Therefore, this study was conducted to examine the contribution of pre-training integrity of the above-mentioned task-switching related white matter fiber groups in predicting task-switching improvement after 12-week TCC exercise training in middle-aged and older adults, by using data collected from a randomized controlled clinical trial (NCT02270320). In particular, we targeted on three task-switching related white matter fiber groups—the prefronto-parietal/occipital group, the PSTP loop group, and the CF group connecting bi-hemispheric prefrontal and parietal cortices (see Methods and Supplementary Table 1). We also included a control fiber group which is not relevant to task-switching—the auditory fiber group of thalamic radiation (TR_auditory_). In light of findings from previous research, we hypothesized that higher pre-training integrity of the three task-switching relevant fiber groups, but not of the control fiber group, would predict greater task-switching improvement after TCC exercise training in middle-aged and older adults. Critically, this prediction should not exist in control participants, who maintained regular daily activities during the study period. We assessed white matter tract integrity using generalized fractional anisotropy (GFA) values as measured with diffusion spectrum imaging (DSI) (Wedeen et al., 2008). We also tested participants' changes in lower extremity strength and walking endurance as physical outcomes. The inclusion of these physical outcome measures afforded reality checks on the physical effects of our TCC training compared to the literature (Lan et al., 2013).

## Methods

### Study Design and Participants

**Figure 1:**
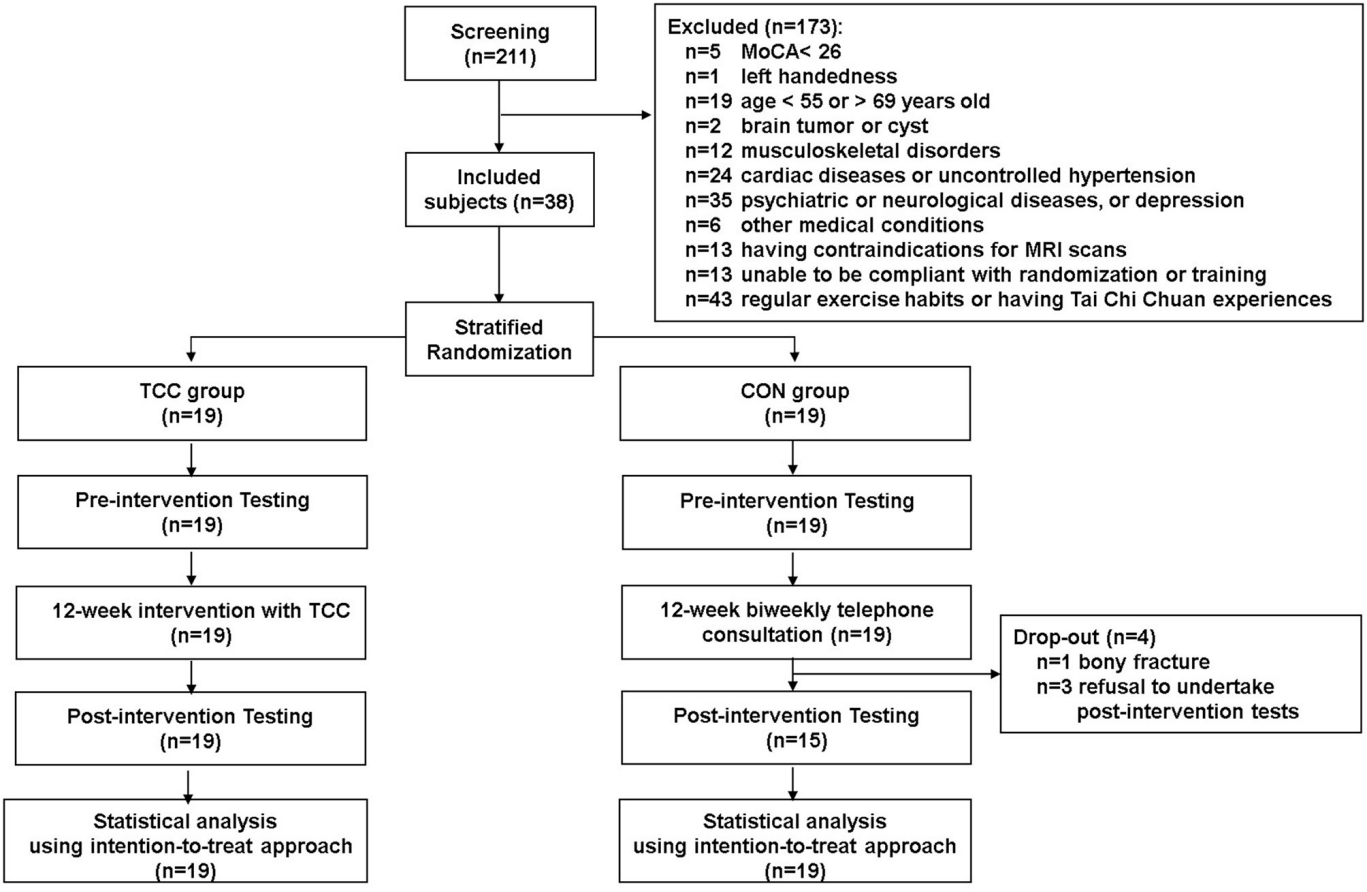
Consort chart of this study.

This is a secondary analysis of data from a registered assessor-blind randomized controlled clinical trial (ClinicalTrials.gov ID: NCT02270320), which examined cognitive effects and neural mechanisms of TCC training in older adults. Participants of the trial were recruited from the local community. The inclusion criteria were being between 50 and 85 years old, education level being >6 years, right-handedness (Oldfield, 1971), being a fluent Mandarin speaker, and having a Montreal Cognitive Assessment Taiwan version score ≧26 (Nasreddine et al., 2005; Tsai et al., 2012) and a Clinical Dementia Rating score = 0 (Hughes et al., 1982). The exclusion criteria were having a Geriatric Depression Scale 15-item short-form (GDS-15) score ≧8 (Nyunt et al., 2009), an Instrumental Activities of Daily Living disability items score ≥1 (Lawton and Brody, 1969), psychiatric or neurological illness, severe or uncontrolled cardiovascular or musculoskeletal diseases, any MRI contraindications, regular moderate-intensity exercise habits (defined as >30 min per session and more than three sessions per week in the past 6 months), and prior experiences with TCC, yoga, qigong, or martial arts. All participants provided written informed consent approved by the Research Ethics Committee of the National Taiwan University Hospital (No. 20121216RIND). Eligible participants were stratified by age (<65 vs. ≧65 years old) and randomly assigned to the TCC or control (CON) group. Demographic information of all participants, including age, sex, cardiovascular risks, systolic blood pressure, diastolic blood pressure, education year, body mass index, and scores on global cognitive function, depression status, and daily physical activity level were also collected before training. Cognitive and physical functions, and neuroimaging data were acquired before and after training, but only the neuroimaging data acquired before training were used in this report. To reduce potential confounding effects from age heterogeneity on outcomes of interest, we used data from participants aged between 55 and 69 years old only in this report. There were 19 in the TCC group and 19 in the control (CON) group (Figure 1). All TCC participants completed the 12-week training and remained enrolled throughout the study. Four CON participants dropped out; one was due to a bone fracture and three were unwilling to complete the post-tests for personal reasons (Figure 1).

### TCC Training and CON Consultation

The TCC training protocol consisted of 1-h group training sessions of the 24-form Yang style TCC (Liang and Wu, 1996) triweekly for 12 weeks. Each session, taught by a certified TCC coach, included a 10-min warm-up, 10-min new-form learning, 30-min sequential form practice, and 10-min cool-down. Participants reached 63–67% of age-predicted maximal heart rate during practice, within the range of moderate intensity based on the ACSM standard (Garber et al., 2011). We also calculated the compliance, defined as percentage of the total sessions attended during the 12-week training period across all TCC participants. The compliance of the TCC group was 100%. Details of the TCC program protocol are described in Wu et al. (2018). The CON participants received one telephone consultation every other week. Except for the TCC group's receiving the 12-week TCC training, both groups of participants were asked not to change their lifestyles or receive other training during the study period in order to ensure good quality control of our study design.

### Task-switching and Physical Function Tests

We used the Intra-Extra Dimensional Set Shift (IED) test from the Cambridge Neuropsychological Test Automated Battery (Cambridge Cognition Ltd., Bottisham, Cambridge, UK) to specifically investigate TCC training effects on task-switching ability. The IED test is a validated computerized analog of the Wisconsin Card Sorting Task (Kim et al., 2014) and has been widely used in research (Stefanova et al., 2014; Chiang et al., 2016). Details about how this test was used in this study can be found in Wu et al. (2018). We used the number of total errors (IED_errors_) among all answered trials to indicate participant's task-switching ability, with a smaller IED_errors_ representing better task-switching ability. Regarding physical functions, we measured participants' muscle strength of bilateral knee extensors using a handheld dynamometer (Lafayette Instrument Co., Lafayette, Indiana, USA) and submaximal functional exercise capacity with the Six-Minute Walk Test (6MWT) (American Thoracic Society Committee on Proficiency Standards for Clinical Pulmonary Function Laboratories, 2002).

### MRI Data Acquisition

MRI was conducted on a 3-Tesla Trio MRI scanner using a 32-channel phased-array head coil (Siemens Healthcare, Erlangen, Germany) at the National Taiwan University Hospital. The T1-weighted images, mainly for subsequent registration of DSI in standard stereotactic space, were acquired using a 3D Magnetization-Prepared Rapid Acquisition Gradient Echo sequence with repetition time (TR)/echo time (TE) = 2,000/2.98 ms, flip angle = 9°, field of view (FOV) = 192 × 256 mm^2^, coronal slice number = 208, and voxel size = 1 × 1 × 1 mm^3^. The DSI was acquired using a single-shot spin-echo echo planar imaging sequence with a twice-refocused balanced echo (Reese et al., 2003), with TR/TE = 9,600/130 ms, flip angle = 90°, FOV = 200 × 200 mm^2^, matrix size = 80 × 80, slice number = 56, and slice thickness = 2.5 mm. The acquisition scheme of DSI consisted of 102 diffusion-weighted image volumes (101 diffusion gradient vectors + 1 null image), corresponding to the grid points in a half sphere of the q-space with the maximum diffusion b-values (b_max_) equal to 4,000 s/mm^2^ (Kuo et al., 2008). The total scan time approximated 20 min.

### Reconstruction of DSI Data

First, we used a quality assurance pipeline to ensure image quality of the entire dataset (Chen et al., 2015). We then transformed the qualified DSI data to obtain the probability density function based on the Fourier relationship between the probability density function and q-space signal (Callaghan et al., 1991). Subsequent processes involved DSI reconstruction and computation of the orientation distribution function (Chiang et al., 2016). The generalized fractional anisotropy (GFA) at each voxel was quantified as the ratio between the standard deviation and root mean square of orientation distribution function. Thus, GFA ranges from zero (indicating completely isotropic) to one (indicating diffusion being restricted to only one direction) and specifies the directionality of the orientation distribution function (Tuch, 2004; Fritzsche et al., 2010). In our study, we interpret greater GFA values to indicate better white matter tract integrity.

### Tract-specific Sampling of GFA Values

Tract-based automatic analysis was used to reconstruct 76 white matter tracts of the whole brain in each participant (Chen et al., 2015). We used the tract atlas developed by Chen et al. (2015), who adopted the automated anatomical labeling system from WFU Pickatlas (version 3.0.4) to obtain cortical and subcortical regions of interests in the Montreal Neurobiology Institute template that were transferred to the NTU-DSI-122 template (please access http://abmri.mc.ntu.edu.tw/tractatlas/TBAA_tractatlas.html from IE for details of the DSI template and tract atlas). Specifically, the T1-weighted and DSI images of study participants were registered to form a study-specific template and served as the input to the tract-based automatic analysis (Chen et al., 2015). The study-specific template was further registered to the standard DSI template NTU-DSI-122 (Hsu et al., 2015) which established the spatial transformation between NTU-DSI-122 and individual DSI images. The coordinates of white matter tracts built in the DSI template were then back-transformed from the template to individual DSI datasets, and GFA values were sampled along the coordinates of each white matter tract automatically for each participant's DSI data.

**Figure 2:**
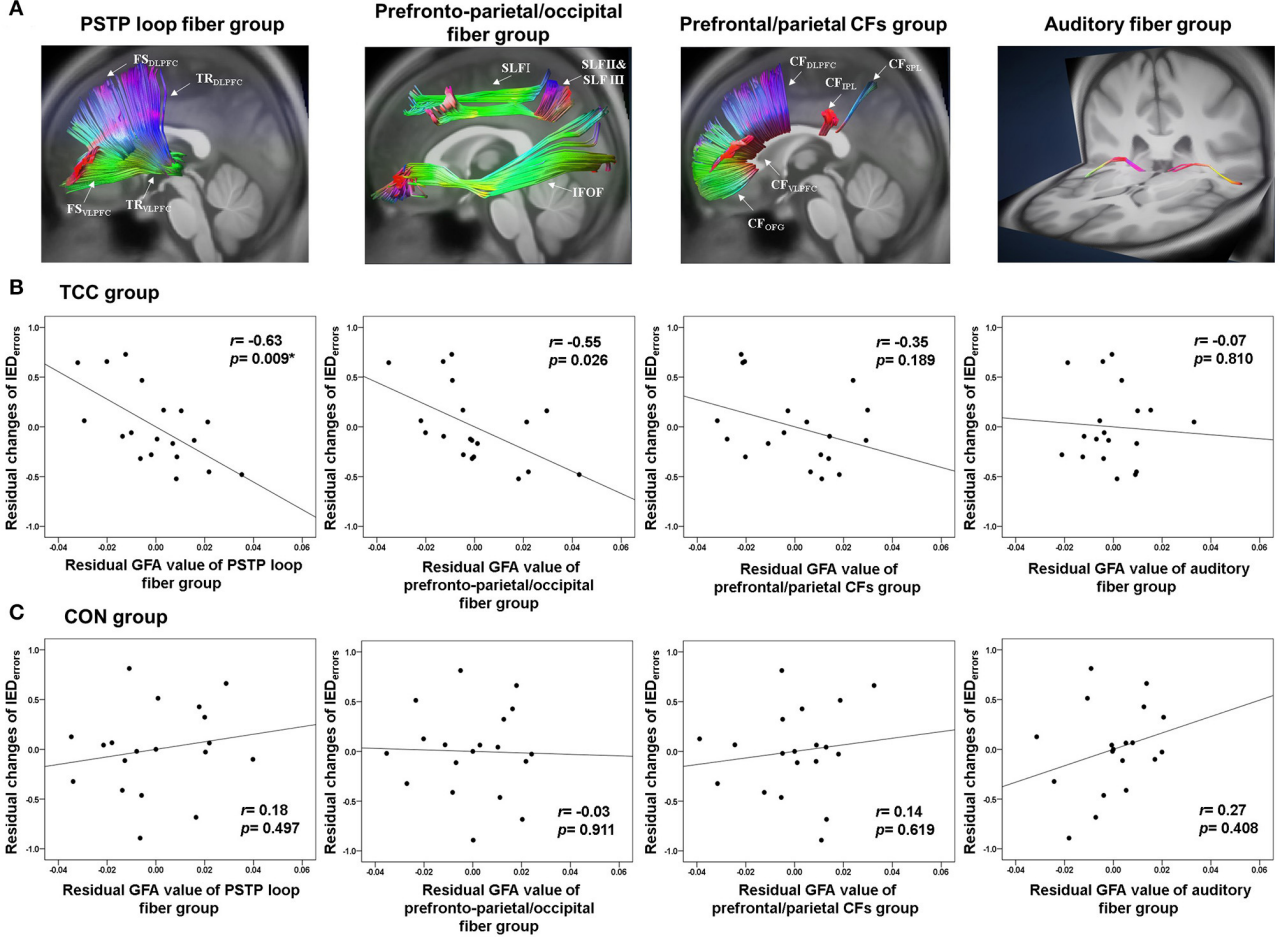
**(A)** Reconstruction of the PSTP loop, prefronto-parietal/occipital, prefrontal/parietal CFs, and auditory fiber groups using the TBAA procedure. **(B)** Partial correlation plots of the GFA values of the PSTP loop, prefronto-parietal/occipital, prefrontal/parietal CFs, and auditory fiber groups with normalized changes of the number of total errors of the IED test for the TCC group. **(C)** Partial correlation plots of these four GFA values with normalized changes of the number of total errors of the IED test for the CON group. CFs, callosal fibers; CON, control group; GFA, generalized fractional anisotropy; IED, Intra-Extra Dimensional Set Shift; PSTP, prefronto-striato-thalamo-prefrontal; TCC, Tai Chi Chuan group. *p < 0.0125 (=0.05/4 adjusted): significant correlations.

We then analyzed the GFAs of the three target white matter fiber groups—the prefronto-parietal/occipital fiber group, the PSTP loop fiber group, and the CF group—as well as a control auditory fiber group for all participants (Figure 2A). All fiber groups were applied in bilateral hemispheres (Supplementary Table 1). The prefronto-parietal/occipital fiber group consisted of superior longitudinal fasciculus and inferior fronto-occipital fasciculus fibers. The PSTP loop fiber group consisted of fibers connecting the medial frontal gyrus, superior frontal gyrus, middle frontal gyrus, and inferior frontal gyrus with the striatum, and fibers connecting the thalamus with the medial frontal gyrus, superior frontal gyrus, middle frontal gyrus, inferior frontal gyrus, orbitofrontal gyrus, and supplementary motor area. The CF group included fibers passing the corpus callosum and connecting bilateral prefrontal gyri (medial frontal, superior frontal, middle frontal, inferior frontal, and orbitofrontal) and superior and inferior parietal lobules. We also applied a control white matter fiber group, the TR_auditory_, which included fibers connecting the thalamus and the Heschl's gyrus. This was chosen as the control fiber group because it was expected to be irrelevant to performance on the IED test since no auditory stimuli were used in the test. Such differential predictive power between IED test-relevant and -irrelevant fiber groups could provide important validation of the specificity of neural correlates of task-switching. The average GFA values across all white matter tracts in each fiber group were calculated to indicate the integrity of the fiber group for pre-test DSI data and for both the TCC and control groups.

### Statistical Analyses

We applied an intention-to-treat approach to analyze all data in this study. The pre-test group differences in demographics, general cognitive function, task-switching ability (IED_errors_), physical functions (average of bilateral knee extensor strength and 6MWT), and GFA values of the four white matter fiber groups were analyzed with chi-square tests for categorical data, with independent samples *t*-tests for continuous variables passing the Shapiro-Wilk test for normality, and with the Mann-Whitney U tests for continuous variables violating the assumption of normality. The significance level was set at *p* < 0.05.

To investigate within-group differences from pre-test to post-test and between-group differences at pre- and post-tests on IED_errors_ and the two physical function measures (strength of knee extensors and 6MWT performance), we also first tested the normality of these variables. We then used the Mann-Whitney *U* tests and Wilcoxon signed-rank test for between- and within-group comparisons, respectively, for variables violating the normality assumption; and used the independent samples *t*-test and paired *t*-test for between- and within-group comparisons, respectively, for variables meeting the normality assumption. The significance level was adjusted to *p* < 0.025 (=0.05/2) for IED_errors_ and to *p* < 0.0125 (=0.05/4) for the physical function measures due to multiple comparisons.

Partial correlation analyses were conducted to investigate whether the baseline GFA values of the four white matter fiber groups (three targets, one control) were associated with normalized changes in IED_errors_ after 12 weeks for both groups, controlling for age, education, and sex. In consideration of potential influences from individual baseline IED_errors_ value on these changes, we normalized the changes in IED_errors_ with baseline IED_errors_ (i.e., change in IED_errors_ = [post-test IED_errors_—pre-test IED_errors_]/pre-test IED_errors_), and used these normalized change values in later partial correlation analyses and regression analyses. Significance level was adjusted to *p* < 0.0125 (=0.05/4) due to multiple partial correlations.

Furthermore, a stepwise multiple linear regression analysis was conducted to determine baseline integrity of which fiber group best predicted normalized reductions of IED_errors_ after TCC training, with the significance level set at *p* < 0.05. In the stepwise method, we used the criteria of *p(F)* < 0.05 to enter and *p(F)* ≧ 0.15 to remove potential predictors. All statistical analyses were performed using SPSS Statistics for Windows, version 18.0.

To understand whether the cognitive task-switching improvement of the TCC participants was associated with their physical improvement, we further performed partial correlation analyses of normalized changes in IED_errors_ with normalized changes in knee extensor strength and 6MWT distance, controlling age, sex, and education. All these changes (=post-test—pre-test values) were normalized to each participant's corresponding pre-test scores. Significance level was set at *p* < 0.025 (=0.05/2) for multiple correlation analyses.

## Results

### Baseline Group Differences and Behavioral Improvements

There were no baseline group differences in demographics, cardiovascular risks, blood pressure, general cognitive function, IED_errors_, and physical functions (all *p* > 0.05) (Tables 1, 2). Table 2 shows that the TCC group significantly reduced the IED_errors_ [Wilcoxon signed-rank test, *z* = –2.40, *p* = 0.016], increased knee extensor strength [*t*_(18)_ = −5.79, *p* < 0.001], and improved 6MWT [Wilcoxon signed-rank test, *z* = –3.55, *p* < 0.001] after training, but the CON group did not have any significant changes in outcome measures. Additionally, the TCC group showed greater post-test knee extensor strength than the CON group [*t*_(36)_ = 2.97, *p* = 0.005]. We also found significant correlations between normalized changes in the number of IED_errors_ with normalized changes in knee extensor strength (*r* = −0.572, *p* = 0.021), but not with normalized changes in 6MWT distance (*r* = −0.273, *p* = 0.305).

**Table 1:** Demographics of the TCC and CON groups at baseline.

	**TCC (*n* = 19) Mean (SD)**	**CON (*n* = 19) Mean (SD)**	***p*-Value**
Age (year)	63.6 (4.0)	63.2 (4.4)	0.801
Sex (female: male)^a^	15:4	18:1	0.150
Hypertension (yes: no)^a^	3:16	7:12	0.141
Dyslipidemia (yes: no)^a^	3:16	1:18	0.290
Diabetes type II (yes: no)^a^	1:18	3:16	0.290
Number of cardiovascular risks (0:1:2)^a^	14:3:2	10:7:2	0.322
Cardiovascular drug dosage (tablets/day)^b^	1.5 (0.8)	1.3 (0.8)	0.630
Systolic blood pressure (mmHg)	118.5 (10.2)	118.7 (15.1)	0.950
Diastolic blood pressure (mmHg)	79.0 (11.5)	76.8 (10.2)	0.533
Education (year)^b^	13.6 (2.2)	13.4 (2.4)	0.951
Body mass index (kg/m^2^)	22.6 (2.5)	22.8 (3.3)	0.849
MoCA (score)^b^	28.3 (1.4)	28.5 (1.4)	0.696
GDS-15 (score)^b^	1.5 (1.9)	1.7 (1.5)	0.306
PASE (score)^b^	54.6 (43.5)	58.4 (52.9)	0.965

a Chi-square tests to test between-group differences in sex, hypertension, dyslipidemia, and diabetes.

b *Mann-Whitney U tests to test between-group differences in education, MoCA, GDS-15, and PASE*.

*CON, control group; GDS-15, Geriatric Depression Scale 15-item Short-form; MoCA, Montreal Cognitive Assessment; PASE, Physical Activity Scale for the Elderly; SD, standard deviation; TCC, Tai Chi Chuan group*.

**Table 2:** Behavioral performances of the TCC and CON groups at pre- and post-tests.

	**TCC group**			**CON group**			**Between-group differences at pre-test**	**Between-group differences at post-test**
	**Pre-test** **Mean (SD)**	**Post-test** **Mean (SD)**	**Within-group differences**	**Pre-test** **Mean (SD)**	**Post-test** **Mean (SD)**	**Within-group differences**		
**Task-switching function**								
IED_errors_	27.1 (13.2)	19.2 (10.5)	0.016^*^	25.1 (15.7)	27.2 (14.3)	0.109	0.726	0.090
**Physical functions**								
Knee extensor strength (kg)	22.8 (5.8)	31.9 (9.5)	<0.001^†^	23.1 (4.1)	24.9 (3.9)	0.024	0.977	0.005^§^
6MWT (m)	474.4 (75.0)	518.2 (66.4)	<0.001^‡^	492.1 (49.7)	490.2 (51.7)	0.795	0.399	0.109

*6MWT, Six-Minute Walk Test; CON, control group; IED_errors_, total errors of Intra/Extra-dimensional set shift test; SD, standard deviation; TCC, Tai Chi Chuan group*.

* *p < 0.025: significant within-group differences between pre- and post-test IED_errors_ using Wilcoxon signed-rank tests for the TCC group*.

† *p < 0.0125: significant within-group differences between pre- and post-test knee extensor strength using paired t-test for the TCC group*.

‡ *p < 0.0125: significant within-group differences between pre- and post-test 6MWT using Wilcoxon signed-rank test for the TCC group*.

§ *p < 0.0125: significant between-group differences in post-test knee extensor strength using independent t-test*.

### Relationships Between Baseline White Matter Integrity and Task-switching Improvement

Partial correlation analyses revealed that in the TCC group, higher baseline GFA of the PSTP loop fiber group (*r* = −0.63, *p* = 0.009), but not of the prefronto-parietal/occipital (*r* = −0.55, *p* = 0.026), CF (*r* = −0.35, *p* = 0.189), and auditory fiber (*r* = −0.07, *p* = 0.810) groups, was associated with greater normalized reductions of the IED_errors_ (Figure 2B; Supplementary Table 2). In the CON group, baseline GFAs of all four fiber groups were not significantly associated with changes in normalized IED_errors_ (*r* = −0.03~0.27, *p* > 0.1) (Figure 2C). Therefore, regression analysis was performed for the TCC group only, with age, sex, and education being the covariates and baseline GFAs of the PSTP loop and prefronto-parietal/occipital fiber groups being the potential predictors for the reduction of IED_errors_ (Table 3). The final model revealed that greater baseline GFA value of the PSTP loop [standardized coefficient (β) = −0.620, *p* = 0.009] significantly predicted greater normalized reduction of IED_errors_ after TCC training, after controlling for age (β = −0.547, *p* = 0.018), sex (β = −0.713, *p* = 0.001), and education (β = −0.491, *p* = 0.018) (Table 3). The negative sign for all β values of the final model indicated that older age, being female, higher education, and better baseline integrity of the PSTP loop significantly predicted greater normalized reduction of IED_errors_ (Adjusted R^2^ = 0.507 for the final model). Baseline GFA of the PSTP loop alone accounted for 26.5% of the variance of changes in IED_errors_ and was the only white matter integrity predictor of normalized reductions in IED_errors_ after TCC training (Table 3).

**Table 3:** Final model of multiple linear regression analysis results on predictors of the normalized changes of IED_errors_ after TCC training.

	**Unstandardized coefficients**		**Standardized coefficients**		**Adjusted R^**2**^**	**Adjusted R^**2**^ change**	***F***	***p*-Value**
	***B***	**Standard error**	**Beta**	***p-*Value**				
Constant	13.560	3.663		0.002^*^				
**Covariates**								
Age	−0.066	0.025	−0.547	0.018^*^				
Sex	−0.831	0.208	−0.713	0.001^*^				
Education	−0.107	0.040	−0.491	0.018^*^				
GFA_pre_ of the PSTP loop	−13.871	4.610	−0.620	0.009^*^	0.507	0.265	9.054	0.009^*^

*GFA_pre_, generalized fractional anisotropy at baseline; IED_errors_, the number of total errors of the Intra/Extra-dimensional set shift test; PSTP, prefronto-striato-thalamo-prefrontal; TCC, Tai Chi Chuan group. Normalized change of IED_errors_ = (post-test IED_errors_ – pre-test IED_errors_)/pre-test IED_errors_*.

*The stepwise method was applied, using the criteria of p(F) < 0.05 as the probability-to-enter and p(F) ≧ 0.10 as probability-to-remove. Age, sex, and education were entered as covariates. The GFA_pre_ values of the PSTP loop and prefronto-parietal/occipital fiber group were originally entered into the regression analysis, but the GFA_pre_ of the prefronto-parietal/occipital fiber group was removed in the final model based on the set criteria*.

* *p < 0.05*.

Since most of the participants in this study were females, we further analyzed the data by using females' data only (*N* = 33; 15 in the TCC group and 18 in the CON group) (Supplementary Table 3) to understand whether the results remained similar. The statistical results of data from female participants turned out to be very similar to the results of *N* = 38. The TCC group significantly reduced the number of IED_errors_ after training, but not the CON group (*p* = 0.01) (Supplementary Table 4). Partial correlation results also revealed that in the TCC group, only baseline integrity of the PSTP tracts significantly correlated with greater reductions in the number of IED_errors_ (*r* = −0.565, *p* = 0.044). Results of the stepwise regression analysis for the TCC group revealed that baseline GFA of the PSTP loop was the only white matter integrity predictor of normalized reductions of IED_errors_ after TCC training (β = −0.748, *p* = 0.044) and alone accounted for 31.3% of the variance of changes in IED_errors_ (Supplementary Table 5).

## Discussion

Our results showed that middle-aged and older adults undergoing 12-week TCC training presented significant task-switching and physical function improvements. More importantly, our hypothesis that pre-training integrity of task-switching relevant white matter fiber groups could predict cognitive task-switching improvement after TCC exercise training in these adults was partially supported. Specifically, better pre-training integrity of the PSTP loop fiber group predicted greater reductions of task-switching errors after TCC exercise training and baseline integrity of the PSTP loop fiber group accounted for more than a quarter of the variance in task-switching improvement, after adjusting for important demographic factors. These novel findings highlight the important influence of pre-existing integrity of prefronto-striatal white matter circuits on the gains of cognitive task-switching ability from TCC training in older adults. The findings that integrity of the other chosen fibers did not significantly predict task-switching improvement after TCC training deviated from our expectations and suggested that practicing TCC may predominantly facilitate the switching between sequential forms of TCC via engaging the PSTP loop fiber group but not the other chosen fiber groups.

Consistent with previous studies, the significant reductions of IED_errors_ in our TCC participants provide additional support that 12-week TCC training was sufficient to induce detectable task-switching improvement in community-dwelling sedentary older adults (Matthews and Williams, 2008; Wu et al., 2018). We speculated that such task-switching effects may come from the combined *mind* and *body* aspects of training of TCC exercises. Past research has shown that physical exercises, such as aerobic and resistance training, improve executive functions in older adults (Colcombe and Kramer, 2003; Liu-Ambrose et al., 2010; Smith et al., 2010). Our TCC participants also improved significantly on knee extensor strength and 6MWT performance (Lan et al., 2013). Therefore, it is possible that the task-switching effects may partially be attributable to the resistance and aerobic effects of TCC exercises. However, we should note that the association between improved knee extensor strength and improved cognitive task-switching cannot be interpreted as a causal relationship. Also, we did not observe significant associations between normalized changes in 6MWT and IED_errors_. As such, we speculate that task-switching effects could alternatively be ascribed to the *mind* aspect of training embedded in TCC, in which remembering movement sequences and proper switching between sequential forms are constantly challenged, which is more directly linked to cognitive task-switching processing. That being said, it remains difficult to determine to what extent task-switching effects were due to the *mind* or *body* aspect of TCC training given the current design in our study. Further studies that include an active control involving another form of physical exercise and another active control involving mental imagery of TCC sequences without physical practice are warranted to tease out the causal contributions of *mind* and *body* aspects of TCC training toward cognitive task-switching improvement.

Importantly, the nature of TCC practice necessarily emphasizes mind-body integration, concentration, and repeated switching between sequential forms. As such, white matter circuits commonly needed for motor and cognitive task-switching and sequencing must be engaged during TCC practice, which, over extended engagement, should theoretically lead to both motor and cognitive task-switching improvements. Indeed, our finding of the predictive power of baseline integrity of the PSTP loop not only supports its role in motor sequence learning (Bennett et al., 2011; Song et al., 2012), but also suggests that this loop is a distinct and important neural circuit for processing task-switching (Ravizza et al., 2012; Klanker et al., 2013).

Many MRI studies have noted the importance of the PSTP loop for task-switching performance (Cools et al., 2004; Coxon et al., 2010; Ystad et al., 2011; van Schouwenburg et al., 2015; Zhu et al., 2015; Serbruyns et al., 2016). Functional MRI studies on young adults show concurrent activation in the prefrontal cortex and basal ganglia (caudate, putamen, and pallidum) during task-switching (Coxon et al., 2010; van Schouwenburg et al., 2015). Such prefronto-striatal pathway involvement in task-switching is perhaps largely due to its roles in action programming, inhibitory control, and decision making (Aron and Poldrack, 2006; Neubert et al., 2010; Ratcliff and Frank, 2012). Aron and Poldrack (2006) conducted a Stop-signal fMRI task and found that the prefronto-striatal pathway processing was involved in response inhibitory control. Using neurocomputational and diffusion models, Ratcliff and Frank (2012) found that the cortico-subcortical pathway connecting the prefrontal and basal ganglia/subthalamic nucleus was involved in decision-making processes to modulate conflicts. Both inhibitory control and decision making are cognitive processes required in performing task-switching. Furthermore, striatal dopamine function is also implicated in the regulation of task-switching performance (Klanker et al., 2013). Patients with Parkinson's disease have dopamine depletion and metabolic dysregulation in prefronto-striatal pathways and typically show task-switching deficits (Cools et al., 2001; Chu et al., 2019). Greater abnormality in the prefrontal white matter tracts was correlated with poorer Stroop performances in these patients (Lenka et al., 2020).

Taken together, it is likely that the form switching and motor sequence learning processes during TCC practice are both facilitated by higher baseline integrity of the PSTP loop. Perhaps what is remarkable in our study is that a predominantly motor sequence ability drilled in TCC practice transfers to cognitive task-switching ability, which is not explicitly trained. We speculate that this might be because PSTP loop integrity also has implications for general efficiency of cortical-subcortical communication for selecting appropriate actions to meet desired goals. Cortical-subcortical information exchange becomes critical when goals switch, and so actions must also switch. Indeed, previous fMRI studies showed that, after TCC training, older adults who had greater increases in resting-state low frequency functional activations or connectivity within the prefrontal regions (Li et al., 2014; Yin et al., 2014), or those who had greater increases in prefrontal activations during task-switching (Wu et al., 2018), showed better task-switching improvement. Overall, we conjecture that enhanced prefrontal activations or local connectivity after TCC training may benefit from better PSTP loop integrity, and therefore lead to greater task-switching improvement. This speculation warrants further testing.

It should be noted that the finding that baseline integrity of the prefronto-parietal/occipital fiber group was not significantly related to IED_errors_ reductions after TCC training did not support our hypothesis. One possible explanation is that the prefronto-parietal/occipital fiber group may be less involved in specific switching processing, albeit its involvement in more general aspects of motor information processing (Rodriguez-Herreros et al., 2015), visuo-spatial attention (Bennett et al., 2012), working memory (Rizio and Diaz, 2016), as well as integration of perception to action (Seghier, 2013). Since our TCC participants were TCC beginners, they had to pay close attention to their own and the coach's body movements during TCC practices, by integrating the constantly changing visuo-spatial and somatosensory information. Better white matter integrity of the prefronto-parietal/occipital fiber group may have helped these participants to have deeper prefrontal, parietal, and occipital engagement and better processing of visuo-spatial attention and working memory, but not better task-switching. Studies targeting and manipulating more specific facets of TCC training and examining their effects are needed to pinpoint the precise role of the prefronto-parietal/occipital fiber group.

Additionally, the finding that baseline integrity of the prefrontal/parietal CFs was not associated with task-switching improvement after TCC training also deviated from our expectations based on previous literature (Madden et al., 2009). We speculate that the lack of predictive power of the prefrontal/parietal CFs for task-switching improvement in this study may be due to our inclusion of a wider range of CF fibers and use of different task-switching performance measures and a prospective research design rather than a cross-sectional design, compared to the previous study. The auditory fiber group was the control fiber group and was not expected to be predictive of task-switching improvement, which was supported in our findings.

One important clinical implication from our findings is that with the same intensity of short-term (12 weeks) TCC training, middle-aged and older individuals with poorer structural integrity of the PSTP pathway at the beginning of training may not benefit from short-term TCC training for improving task-switching ability as much as those with better baseline structural integrity of this pathway. These individuals may include people with multiple cardiovascular risks, patients with Parkinson's disease and psychosis (Lenka et al., 2020), or patients with small vessel disease who already have severe white matter hyperintensities (Zhu et al., 2019). The presence of cardiovascular risks is negatively associated with brain white matter health in not only middle-aged and older adults (Cox et al., 2019) but also young adults (Williamson et al., 2018). In a large-scale cohort study on middle-aged and older adults, Cox et al. (2019) found that those with a greater number of cardiovascular risks had poorer brain white matter microstructure, particularly in the thalamic radiations. Indeed, when we further compared GFA values of the PSTP loop of our participants with cardiovascular risks of hypertension, dyslipidemia, or diabetes and those without these risks, we found that the GFA value of the former sub-group (0.452) was significantly lower than that of the latter subgroup [0.476; *F*_(1,33)_ = 0.982, *p* = 0.007, controlling for age, sex, and education]. For middle-aged and older adults without any cardiovascular risks, small vessel disease, and Parkinson's disease, our findings highlight the importance of maintaining brain white matter integrity from young adulthood (Williamson et al., 2018), perhaps through a healthy lifestyle that prevents cardiovascular risks, in order to gain better exercise training effects on cognitive functions.

In patients with cerebral small vessel disease and patients with Parkinson's disease, abnormality in serum inflammatory and lipoprotein markers, including trefoil factor 3, cholinesterase activity, homocysteine, lipoprotein cholesterol, lipoprotein-associated phospholipase A2, and superoxide dismutase, have been reported to be predictive of cognitive impairment or disease severity (Zou et al., 2018; Ahmadi Rastegar et al., 2019; Zhu et al., 2019; Wang et al., 2020; Yang et al., 2020). Although we did not measure the changes of these serum markers in this study, it might be possible that patients with greater abnormalities in these serum biomarkers also would show poorer responses to TCC training for cognitive benefits.

Taken together, it is worth further investigating whether individuals who have only mild white matter tract degradations, such as those with a single cardiovascular risk or *de novo* stage Parkinson disease (Ni et al., 2014; Taylor et al., 2018), could benefit from a longer-term (>6 months) of TCC training for improving their task-switching ability and delaying the declines in white matter structural integrity. Future studies investigating the interrelationships among cardiovascular risks, pathogenesis and biomarkers of neurodegenerative diseases, brain white matter integrity, and exercise-related benefits of cognition are also warranted.

### Limitations

We acknowledge that small sample size, the sample being skewed toward females, and lack of an active control are three limitations of this study. This study had a relatively small sample size of mostly women of more homogeneous age range. Thus, results of our findings need to be interpreted with caution and can only be applied to middle-aged and younger older adults, especially females. Our finding that female sex predicted greater improvement in task-switching after TCC practice also needs to be interpreted with caution. Replications of this study using a larger sample size with a more balanced ratio between number of males and females are warranted. Including an active control group, either engaging in another type of physical exercise or engaging in mental practices of TCC sequences, would provide more comprehensive understanding of the causal relationship of *mind* and *body* components of TCC practices on cognitive task-switching. Despite these limitations, this carefully conducted randomized controlled trial has provided an important novel finding that baseline integrity of the PSTP loop fibers could predict cognitive task-switching improvement after TCC training in middle-aged and older adults.

## Conclusions

This is the first study investigating how baseline integrity of brain white matter tracts may influence cognitive effects of TCC training in middle-aged and older adults. Findings of this study partially supported our hypothesis by showing that pre-training integrity of the PSTP loop fibers predicted task-switching improvement after 12 weeks of TCC exercise training in middle-aged and older adults. These findings suggest that TCC training may predominantly engage the PSTP loop fibers to perform the repetitive, sequential form-switching tasks. Thus, better baseline PSTP loop integrity may facilitate more effective TCC training and allows for better transfer of the motor switching ability to cognitive switching ability.

## Data Availability Statement

The datasets generated or analyzed in this study are available from the corresponding author on reasonable request. Requests to access the datasets should be directed to Pei-Fang Tang, pftang@ntu.edu.tw.

## Ethics Statement

All procedures performed in studies involving human participants were in accordance with the ethical standards of the Research Ethics Committee of the National Taiwan University Hospital (No. 20121216RIND) and with the 1964 Helsinki declaration and its later amendments or comparable ethical standards. Informed written consents were obtained from all participants.

## Author Contributions

M-TW and P-FT contributed to the conception of the work, completed the first draft, and final version of the manuscript. M-TW, P-FT, W-YIT, JOSG, Y-KC, T-LC, SS-G, and CL contributed to the design of the work. M-TW, W-YIT, Y-CH, and Y-JC contributed to the data acquisition and analysis. M-TW, P-FT, JOSG, W-YIT, Y-KC, Y-CH, Y-JC, T-LC, SS-G, and CL contributed to interpretation of data. All authors were involved in the manuscript revision and agreed with final approval of the version, and ensured the accuracy of investigation.

## Conflict of Interest

The authors declare that the research was conducted in the absence of any commercial or financial relationships that could be construed as a potential conflict of interest.
