# Supplementary material for: Integrity of the Prefronto-striato-thalamo-prefrontal Loop Predicts Tai Chi Chuan Training Effects on Cognitive Task-switching in Middle-aged and Older Adults
Source: Front Aging Neurosci. 2021 Feb 15;12:602191. doi: 10.3389/fnagi.2020.602191 (PMC7917054; doi:10.3389/fnagi.2020.602191)
Supplement: Supplementary file 1 [file Table_1.pdf]

Supplementary Table 1. Names of fiber tracts included in the four white matter fiber groups and their connected ROIs

Names of fiber tracts	Connected ROIs	Connected ROIs
**PSTP loop fiber group**		
1. L FS_{DLPFC}	L striatum	L medial frontal gyrus + superior frontal gyrus
2. L FS_{VLPFC}	L striatum	L inferior frontal gyrus + middle frontal gyrus
3. L TR_{DLPFC}	L thalamus	L medial frontal gyrus + superior frontal gyrus + SMA
4. L TR_{VLPFC}	L thalamus	L orbitofrontal gyrus + middle frontal gyrus + inferior frontal gyrus
5. R FS_{DLPFC}	R striatum	R medial frontal gyrus + superior frontal gyrus
6. R FS_{VLPFC}	R striatum	R inferior frontal gyrus + middle frontal gyrus
7. R TR_{DLPFC}	R thalamus	R medial frontal gyrus + superior frontal gyrus + SMA
8. R TR_{VLPFC}	R thalamus	R orbitofrontal gyrus + middle frontal gyrus + inferior frontal gyrus
**Prefronto-parietal/occipital fiber group**		
1. L SLF I	L superior frontal gyrus	L precuneus
2. L SLF II	L inferior frontal gyrus pars triangularis	L middle occipital gyrus
3. L SLF III	L inferior frontal gyrus pars opercularis	L angular gyrus
4. L IFOF	L orbitofrontal gyrus	L occipital lobe
5. R SLF I	R superior frontal gyrus	R precuneus
6. R SLF II	R inferior frontal gyrus pars triangularis	R middle occipital gyrus
7. R SLF III	R inferior frontal gyrus pars opercularis	R angular gyrus

8.	R IFOF	R orbitofrontal gyrus	R occipital lobe
**Prefronto/parietal CFs group**			
1.	CF_{OFG}	L orbitofrontal gyrus	R orbitofrontal gyrus
2.	CF_{DLPFC}	L medial frontal gyrus + superior frontal gyrus	R medial frontal gyrus + superior frontal gyrus
3.	CF_{VLPFC}	L inferior frontal gyrus + middle frontal gyrus	R inferior frontal gyrus + middle frontal gyrus
4.	CF_{SPL}	L superior parietal lobules	R superior parietal lobules
5.	CF_{IPL}	L inferior parietal lobules	R inferior parietal lobules
**Reference fiber group: Auditory fiber group**			
1.	L $TR_{auditory}$	L thalamus	L Heschl's gyrus
2.	R $TR_{auditory}$	R thalamus	R Heschl's gyrus

The table is adapted from Chen et al. (2015). Abbreviations: CFs, callosal fibers; CST, corticospinal tract; DLPFC, dorsolateral prefrontal cortex; FS, fronto-striatal; IFOF, inferior fronto-occipital fasciculus; IPL, inferior parietal lobules; L, left; OFG, orbitofrontal gyrus; PSTP, prefronto-striato-thalamo-prefrontal; R, right; ROIs, regions of interest; SLF, superior longitudinal fasciculus; SPL, superior parietal lobules; SMA, supplementary motor area; TR, thalamic radiation; VLPFC, ventrolateral prefrontal cortex.
