# Supplementary material for: Integrity of the Prefronto-striato-thalamo-prefrontal Loop Predicts Tai Chi Chuan Training Effects on Cognitive Task-switching in Middle-aged and Older Adults
Source: Front Aging Neurosci. 2021 Feb 15;12:602191. doi: 10.3389/fnagi.2020.602191 (PMC7917054; doi:10.3389/fnagi.2020.602191)
Supplement: Supplementary file 2 [file Table_2.pdf]

Supplementary Table 2. Partial correlations of the GFA values of the PSTP loop, prefronto-parietal/occipital, prefrontal/parietal CFs, and auditory fiber groups with normalized changes of the number of total errors of the IED test for the TCC and CON groups

	PSTP group	Prefronto-parietal/occipital tracts	Prefrontal/parietal CFs	Auditory tracts
TCC group	r= -0.63	r= -0.55	r= -0.35	r= -0.07
	p= 0.009*	p= 0.026	p= 0.189	p= 0.810
CON group	r= 0.18	r= -0.03	r= 0.14	r= 0.27
	p= 0.497	p= 0.911	p= 0.619	p= 0.408

CFs, callosal fibers; CON, control group; GFA, generalized fractional anisotropy; IED, Intra-Extra Dimensional Set Shift; PSTP, prefronto-striato-thalamo-prefrontal; TCC, Tai Chi Chuan group. * $p < 0.0125$ (= 0.05/4 adjusted): significant correlations.
