# Supplementary material for: Integrity of the Prefronto-striato-thalamo-prefrontal Loop Predicts Tai Chi Chuan Training Effects on Cognitive Task-switching in Middle-aged and Older Adults
Source: Front Aging Neurosci. 2021 Feb 15;12:602191. doi: 10.3389/fnagi.2020.602191 (PMC7917054; doi:10.3389/fnagi.2020.602191)
Supplement: Supplementary file 3 [file Table_3.pdf]

Supplementary Table 3. Demographics of the TCC and CON groups at baseline for the female participants (n=33)

	TCC (n = 15) mean (SD)	CON (n = 18) mean (SD)	*p*-value
Age (year)	63.4 (4.3)	63.4 (4.4)	0.972
Sex (female : male)	15:0	18:0	-
Hypertension (yes : no) [a]	2:13	6:12	0.182
Dyslipidemia (yes : no) [a]	2:13	1:17	0.439
Diabetes type Ⅱ (yes : no) [a]	1:14	3:15	0.381
Number of CVD risks (0:1:2) [a]	11:3:1	10:6:2	0.572
CVD drug dosage (tablets/day) [b]	0.4 (0.7)	0.6 (0.9)	0.323
SBP (mmHg)	118.7 (8.8)	118.3 (15.5)	0.942
DBP (mmHg)	78.9 (11.9)	76.2 (10.1)	0.484
Education (year) [b]	13.3 (2.2)	13.4 (2.5)	0.686
Body mass index (kg/m^2)	22.8 (2.6)	22.7 (3.3)	0.950
MoCA (score) [b]	28.3 (1.5)	28.6 (1.3)	0.602
GDS-15 (score) [b]	1.5 (1.7)	1.7 (1.5)	0.503
PASE (score) [b]	53.0 (41.7)	51.1 (43.8)	0.928

[a] Chi-square tests to test between-group differences in sex, CVD risks, hypertension, dyslipidemia, and diabetes.

[b] Mann-Whitney U tests to test between-group differences in education, MoCA, GDS-15, and PASE.

CON, control group; CVD, cardiovascular disease; DBP, diastolic blood pressure; GDS-15, Geriatric Depression Scale 15-item Short-form; MoCA, Montreal Cognitive Assessment; PASE, Physical Activity Scale for the Elderly; SBP, systolic blood pressure; SD, standard deviation; TCC, Tai Chi Chuan group.
