# Supplementary material for: Integrity of the Prefronto-striato-thalamo-prefrontal Loop Predicts Tai Chi Chuan Training Effects on Cognitive Task-switching in Middle-aged and Older Adults
Source: Front Aging Neurosci. 2021 Feb 15;12:602191. doi: 10.3389/fnagi.2020.602191 (PMC7917054; doi:10.3389/fnagi.2020.602191)
Supplement: Supplementary file 4 [file Table_4.pdf]

Supplementary Table 4. Behavioral performances of the TCC and CON groups at pre- and post-tests for the female participants (n=33)

	TCC group (n=15)			CON group (n=18)			Between-group differences at pre-test	Between-group differences at post-test
	Pre-test mean (SD)	Post-test mean (SD)	Within-group differences	Pre-test mean (SD)	Post-test mean (SD)	Within-group differences		
**Task-switching function**								
IED_{errors}	27.9 (13.2)	17.6 (9.8)	0.010*	25.8 (15.8)	28.2 (13.9)	0.086	0.717	0.023
**Physical functions**								
Knee extensor strength (kg)	21.4 (5.3)	28.6 (6.4)	<0.001†	22.7 (3.8)	24.6 (3.7)	0.023	0.429	0.030
6MWT (m)	457.4 (65.6)	502.0 (63.0)	0.001‡	489.1 (49.4)	485.1 (48.1)	0.600	0.193	0.286

6MWT, Six-Minute Walk Test; CON, control group; IED_{errors}, total errors of Intra/Extra-dimensional set shift test; SD, standard deviation; TCC, Tai Chi Chuan group.

* $p < 0.025$: significant within-group differences between pre- and post-test IED_{errors} using Wilcoxon signed-rank tests for the TCC group.

† $p < 0.0125$: significant within-group differences between pre- and post-test knee extensor strength using paired-t test for the TCC group.

‡ $p < 0.0125$: significant within-group differences between pre- and post-test 6MWT using Wilcoxon signed-rank test for the TCC group.
