# Supplementary material for: Integrity of the Prefronto-striato-thalamo-prefrontal Loop Predicts Tai Chi Chuan Training Effects on Cognitive Task-switching in Middle-aged and Older Adults
Source: Front Aging Neurosci. 2021 Feb 15;12:602191. doi: 10.3389/fnagi.2020.602191 (PMC7917054; doi:10.3389/fnagi.2020.602191)
Supplement: Supplementary file 5 [file Table_5.pdf]

Supplementary Table 5. Final model of multiple linear regression analysis results on predictors of the normalized changes of IED_{errors} after TCC training for the female participants (n=33)

	Unstandardized coefficients		Standardized coefficients		Adjusted R^2	Adjusted R^2 change	*F*	*p*-value
	B	Standard error	Beta	*p*-value				
Constant	7.733	3.825		0.068				
Covariates								
Age	-0.036	0.027	-0.446	0.207				
Education	-0.063	0.044	-0.405	0.179				
GFA_{pre} of the PSTP loop	-10.393	4.576	-0.748	0.044*	0.152	0.313	5.157	0.044*

GFA_{pre}, generalized fractional anisotropy at baseline; IED_{errors}, the number of total errors of the Intra/Extra-dimensional set shift test; PSTP, prefronto-striatal-thalamo-prefrontal; TCC, Tai Chi Chuan group. Normalized change of IED_{errors} = (post-test IED_{errors} − pre-test IED_{errors}) / pre-test IED_{errors}.

The stepwise method was applied, using the criteria of p(F)< 0.05 as the probability-to-enter and p(F)≧0.10 as probability-to-remove. Age and education were entered as covariates.

$^{*}p < 0.05$.
